# Endothelial Dysfunction Exacerbates Renal Interstitial Fibrosis through Enhancing Fibroblast Smad3 Linker Phosphorylation in the Mouse Obstructed Kidney

**DOI:** 10.1371/journal.pone.0084063

**Published:** 2013-12-31

**Authors:** Yu Bo Yang Sun, Xinli Qu, Xueling Li, David J. Nikolic-Paterson, Jinhua Li

**Affiliations:** 1 Department of Anatomy and Developmental Biology, Monash University, Clayton, Victoria, Australia; 2 The Key Laboratory of National Education Ministry for Mammalian Reproductive Biology and Biotechnology, Inner Mongolia University,Hohhot,Inner Mongolia, People's Republic of China; 3 Department of Nephrology, Monash Health and Monash University Department of Medicine, Clayton, Victoria, Australia; Universidade de Sao Paulo, Brazil

## Abstract

Endothelial dysfunction and enhanced transforming growth factor-β (TGF-β)/Smad3 signalling are common features of progressive renal fibrosis. This study investigated a potential link between these mechanisms. In unilateral ureteric obstruction (UUO) we observed an acute (6 hr) down-regulation of nitric oxide synthase 3 (NOS3/eNOS) levels and increased phosphorylation of the linker region of Smad3 at T179 and S208 in Smad3/JNK complexes. These events preceded Smad3 C-terminal domain phosphorylation and the induction of myofibroblast proliferation at 48 hrs. Mice deficient in NOS3 showed enhanced myofibroblast proliferation and collagen accumulation compared to wild type mice in a 7 day UUO model. This was associated with enhanced phosphorylation of Smad3 T179 and S208 by 92% and 88%, respectively, whereas Smad3-C-terminal phosphorylation was not affected. Resolvin D1 (RvD1) can suppress renal fibrosis in the UUO model, and further analysis herein showed that RvD1 protected against endothelial dysfunction and suppressed Smad3/JNK complex formation with a consequent reduction in phosphorylation of Smad3 T179 and S208 by 78% and 65%, respectively, while Smad3 C-terminal phosphorylation was unaltered. In vitro, conditioned media from mouse microvascular endothelial cells (MMEC) treated with a general inhibitor of nitric oxide synthase (L-NAME) augmented the proliferation and collagen production of renal fibroblasts (NRK49F cells) compared to control MMEC media and this was associated with increased phosphorylation of JNK and Smad3 T179 and S208, whereas Smad3-C-terminal domain phosphorylation was unaffected. The addition of RvD1 to L-NAME treated MMEC abrogated these effects of the conditioned media on renal fibroblasts. Finally, Smad3 T179/V and S208/A mutations significantly inhibit TGF-β1 induced up-regulation collagen I promoter. In conclusion, these data suggest that endothelial dysfunction can exacerbate renal interstitial fibrosis through increased fibroblast proliferation and collagen production via enhanced Smad3 linker phosphorylation.

## Introduction

Renal interstitial fibrosis is the common final pathway in the progression of end stage kidney disease, irrespective of the initial trigger or site of injury. The process of tubulointerstitial fibrosis involves the loss of renal tubules and peritubular capillary endothelial cells, and the accumulation of inflammatory cells, interstitial myofibroblasts and extracellular matrix [1].

The contribution of tubular epithelial cells, fibroblast/myofibroblasts and inflammation to the development of interstitial fibrosis has been extensively studied. However, much less is known about how endothelial cell injury contributes to this process. Recent studies have shown that injury to glomerular endothelial cells directly contributes to podocyte and mesangial cell damage and the development of glomerulosclerosis [2]–[5]. We have shown that injury to glomerular endothelial cells precedes podocyte damage and the development of proteinuria and glomerulosclerosis in adriamycin-induced nephropathy, while nitric oxide synthase 3 (NOS3/eNOS) deficiency accelerates kidney injury in this model [6]. Interesting, conditioned media from NOS3 over-expressing endothelial cells protects cultured podocytes from TNF-α-induced injury [6], prompting us to investigate the potential role of endothelial cell injury on fibroblasts/myofibroblasts in the development of renal interstitial fibrosis.

It is well established that transforming growth factor (TGF)-β1/Smad3 signalling plays an essential role in the development of tissue fibrosis. TGF-β1-induced gene transcription operates via phosphorylation of Smad2 and Smad3 [7]. An important recent finding is that Smad2 and Smad3 play contrasting roles in fibrosis. Conditional gene deletion of Smad2 exacerbates renal fibrosis in the unilateral ureteric obstruction (UUO) model, whereas Smad3 gene deletion (*Smad3-/-*) mice is protective against fibrosis [8], [9]. Indeed, fibroblasts from *Smad3-/-* mice fail to auto-induce TGF-β1 expression [10], and *Smad3-/-* mice are protected in several models of tissue fibrosis (UUO, diabetic nephropathy and angiotensin II-induced renal and cardiac fibrosis), demonstrating a key role for TGF-β/Smad3 signalling in tissue fibrosis [9], [11]–[14]. Phosphorylation is recognised as an important mechanism regulating Smad3 transcription factor activity and thereby the fibrotic response [15]–[18]. The transforming growth factor-β receptor I (TGF-βRI) phosphorylates serine residues in the SSXR motif in the C-terminal domain of Smad3, while mitogen-activated protein kinases (JNK, ERK, p38), protein kinase B (Akt), and cyclin-dependent kinases (CDK4 and CDK8) can phosphorylate residues (T179, S204, S208 and S213) in the Smad3 linker region [19]–[26]. C-terminal phosphorylation is often associated with TGF-β-induced growth inhibition of normal epithelial cells and embryonic fibroblasts and augmentation of TGF-β induced collagen production [27], [28], whereas the role of Smad3 linker region phosphorylation in regulating cell proliferation is controversial [25], [26]. It is unknown whether Smad3 linker region phosphorylation is altered during tissue fibrosis and whether this may be an important regulatory mechanism in either local fibroblast proliferation or collagen production in the pathogenesis of fibrotic disease.

Resolvins belong to a series of naturally occurring lipid-derived mediators that are produced during the resolution of the inflammatory response [29]. The name Resolvin (resolution phase interaction products) was introduced to describe their potent anti-inflammatory and immunoregulatory actions [30]. Resolvins of the E series (Resolvin E1 or RvE1) are derived from eicosapentaenoic acid, while Resolvins of the D series (Resolvin D1 or RvD1) are biosynthesized from the precursor docosahexaenoic acid [30]. The regulatory role of RvE1 in inflammation has been documented in various mouse models of inflammatory disease; including acute peritonitis, acute colitis and periodontitis [31]–[33]. We have established that administration of RvE1 or RvD1 can significantly reduce fibroblast proliferation and extracellular matrix production in the mouse UUO model. In addition, RvE1 was shown to inhibit platelet-derived growth factor-BB (PDGF-BB)-induced proliferation in rat fibroblast NRK49F cells [34]. RvD1 has also been shown to reduce expression of CCL2 and IL-8 in TNF-α activated human aortic endothelial cells [35]. In the current study, we examined whether RvD1 can protect endothelial cells from injury as a mechanism underlying its' anti-proliferative and anti-fibrotic effects in the mouse UUO model.

## Materials and Methods

### Experimental Animals

Wild-type C57BL6/J (8 weeks old) mice were purchased from Monash Animal Services, Monash University, Australia. Breeding pairs of NOS3 gene knockout mice (NOS3-/-) were purchased from Jackson Laboratories (Bar Harbor, ME) and maintained at Monash Animal Services. All experiments were performed with the approval of a Monash University Animal Ethics Committee, which adheres to the “Australian Code of Practice for the Care and Use of Animals for Scientific Purposes.” Groups of 5 male wild-type (WT) or *Nos3-/-* C57BL/6J mice were used in each experiment.

UUO surgery was performed under isofluorane anaesthesia. The left ureter was visualized following a flank incision and ligated with a vascular clamp. Sham mice underwent the same procedure, except that the ureter was not ligated. Mice were killed at 6 hours, 12 hours, 24 hours, 48 hours, 4 days and 7 days after UUO. Kidney tissues were collected for analysis. In addition, we analysed archival tissue from a previous study in which groups of four C57BL/6J mice that underwent UUO surgery and were given tail vein injections of RvD1 (Cayman Chemical, Ann Arbor, Michigan, 4 ng/g every 6 h) or vehicle, beginning on day 2 after UUO and continuing until they were killed on day 4 [34].

### Confocal Microscopy Analysis

Cryostat sections of tissues fixed in 4% paraformaldehyde (Sigma, St. Louis, MO) were blocked with 2% bovine serum albumin in PBS and incubated with the following antibodies: rabbit anti-Ki67 antibody (Abcam, Cambridge, UK) followed by goat anti-Alexa Fluor 488 (Invitrogen, Victoria, Australia) and Cy3-conjugated mouse anti-α-SMA antibody (Sigma); rabbit anti-collagen type I (Biorbyt Ltd, Cambridge, UK) followed by goat anti-rabbit Alexa Fluor 488 (Invitrogen) and rat anti-CD31 (BD Biosciences, San Diego, CA) followed by goat anti-rat Alexa Fluor 488 (Invitrogen). Sections were counterstained with 4,6-diamidino-2-phenylindole (Sigma) to visualize nuclei. Sections were analysed with an Olympus Fluoview 1000 confocal microscope (Olympus, Tokyo, Japan), FV10-ASW software (version 1.3c; Olympus) and oil UPLFL ×60 objective (NA 1.25; Olympus). The number of α-SMA-positive/ki67-positive cells was counted directly. All scoring was performed on blinded slides.

#### Quantification of peritubular capillaries (PTC)

In each sample group, 40 randomly selected high power cortical fields were examined under ×600 magnification for assessment of CD31-positive (+) PTC changes [36]. PTC changes were expressed per mm^2^ as previously described [36].

### Cell Culture

Mouse microvascular endothelial cells (MMEC) were purchased from ATCC (Manassas, VA) and cultured in 5% CO2 atmosphere at 37°C in Dulbecco's modified Eagle's medium (Life Technologies BRL, Gaithersburg, MD) containing 10% fetal bovine serum. MMECs were seeded in 6-well plates at 1×10^6^ cells/well, allowed to adhere overnight and then changed to serum-free media for 4 hours. To examine NOS3 protein expression under inflammatory condition, MMECs were stimulated with recombinant TNF-α (10 ng/ml) with or without RvD1 (4 ng/ml) for 12 hours. Each experiment was repeated at least three times.

### MMEC Conditioned Media

MMECs were separately seeded into 6 well-tissue culture plates at a density of 3×10^6^ cells/well. The cells were incubated for 12 hours then washed three times with PBS prior to fresh media with or without 1 mM Nω-Nitro-L-arginine methyl ester hydrochloride (L-NAME) (Sigma) being added to the cells. The supernatant was collected 24 hours later and is referred to as MMEC media or L-NAME-treated media, respectively. L-NAME and/or low molecular molecules were removed from conditioned media by at least three passes through centrifugal filters with a 10 kDa cut-off (Amicon® Ultra 10K, Merck Millipore, Australia) and then sterile filtered through a 0.22 µM filter.

### Collagen I promoter Luciferase Reporter Assay

HEK293T cells were cultured in 24-well plates. Cells at 80% confluence were transfected with a luciferase reporter construct under control of the −3200 bp to +54 bp of the α2(I) collagen promoter and the PRL-TK control plasmid using Lipofectamine 2000 (maximum of 500 ng total DNA per well) as described previously [37]. Twenty-four hours later the media was replaced with conditioned media from MMEC (control, L-NAME treated cells or L-NAME plus 4 ng/ml RvD1 treated cells) for 18 hours. In separate experiments, HEK293T cells were transfected using Lipofectamine 2000 (maximum of 1.0 µg total DNA per well) with the collagen I promoter luciferase reporter with PRL-TK control plus one of the following plasmids expressing Flag-tagged wild type or mutated Smad3 plus enhanced green fluorescence protein (EGFP): pMSCV-Flag-Smad3 WT-IRES-EGFP, pMSCV-Flag-Smad3 T179/V-EGFP, pMSCV-Flag-Smad3 S208/A-EGFP or pMSCV-Flag-Smad3 3S/A-EGFP. Twenty-four hours later cells were stimulated with or without TGF-β1 for 18 hours. The collagen I promoter activity was examined using the Dual-Luciferase assay kit (Promega, Madison, WI).

### Fibroblast Cell Culture

Rat kidney fibroblast cell line NRK49F cells were purchased from ATCC and grown in a 5% CO_2_ atmosphere at 37°C in DMEM containing 10% fetal bovine serum. Cells were seeded into 6-well culture plates, allowed to adhere overnight, and then changed into serum-free medium for overnight. Cells were cultured in MMEC media or L-NAME-treated media with or without recombinant mouse PDGF-BB (R&D Systems, Minneapolis, MN, 20 ng/ml), TGF-β (R&D Systems, 2 ng/ml) or RvD1 (Cayman Chemical, 4 ng/ml). Each experiment was repeated at least three times.

### Kidney Fibroblast Cell Proliferation Assay

NRK49F cells were seeded into 96-well culture plates or chamber slides, allowed to adhere overnight, and then changed into serum-free media overnight. Cells were stimulated in MMEC media or L-NAME-treated media with or without recombinant mouse PDGF-BB for 24 hours. Cells were fixed in 4% paraformaldehyde (Sigma), blocked with 2% bovine serum albumin in PBS and incubated with rabbit anti-Ki67 antibody (Abcam) followed by goat anti-rabbit conjugated with HRP (Sigma). The number of ki67-positive cells was counted directly. All scoring was performed on blinded slides. Cell proliferation was quantified BrdU Proliferation Kit (Roche Applied Sciences, Castle Hill NSW, Australia).

### Immunoprecipitation and Western Blotting

Kidney tissues and cell culture samples were sonicated and lysed in 0.4 ml RIPA lysis buffer. The tissue and cell extracts were centrifuged at 15,000 rpm and 4°C for 30 minutes to remove cell debris. The protein concentrations were measured by modified Lowry protein assay using BSA as a protein standard (Bio-Rad, Gladesville, New South Wales, Australia). Samples of tissue or cell lysates (1 mg) were immunoprecipitated with mouse anti-Smad3 antibody (Santa Cruz Biotechnology, Dallas, TX) then isolated using protein A/G agarose beads (Santa Cruz Biotechnology). Proteins were electrophoresed through a 10% SDS-PAGE gel before transfer to a PVDF membrane. After blocking for 30 minutes at 4°C in blocking buffer (5% BSA in PBS with 0.1% Tween 20), the membrane was incubated overnight with rabbit anti-eNOS, rabbit anti-fibronectin (Santa Cruz Biotechnology), rabbit anti-C-myc, rabbit anti-p-JNK1/2 and rabbit anti-JNK1/2 (Cell Signaling Technology, San Francisco, CA), rabbit anti-Smad3 p-T179 (Abcam), mouse anti-p21 (Invitrogen), rabbit anti-collagen I (Biorbyt Ltd), mouse anti-α-SMA antibody (Sigma) or rabbit anti-Smad3 p-S208 (Santa Cruz Biotechnology). The membrane was washed and incubated for 30 minutes at room temperature with secondary antibody conjugated with HRP. After further washing, the membrane was detected with ECL kit (Amersham Pharmacia Biotech, Arlington, IL, USA). α-tubulin, GAPDH and Smad3 were used as internal controls. Western blotting images were captured by Kodak 4000 mm and density of the bands was quantitated by using ImageJ (http://rsb.info.nih.gov/ij/).

### Statistical Analysis

Data are shown as mean ±SD, with statistical analyses performed using one-way analysis of variance (ANOVA), with post hoc analysis with Tukey's multiple comparison test using GraphPad Prism 6.0 (Graph-Pad Software, San Diego, CA, USA).

## Results

### Endothelial Dysfunction is an Early Event in the Obstructed Kidney

**Figure 1:**
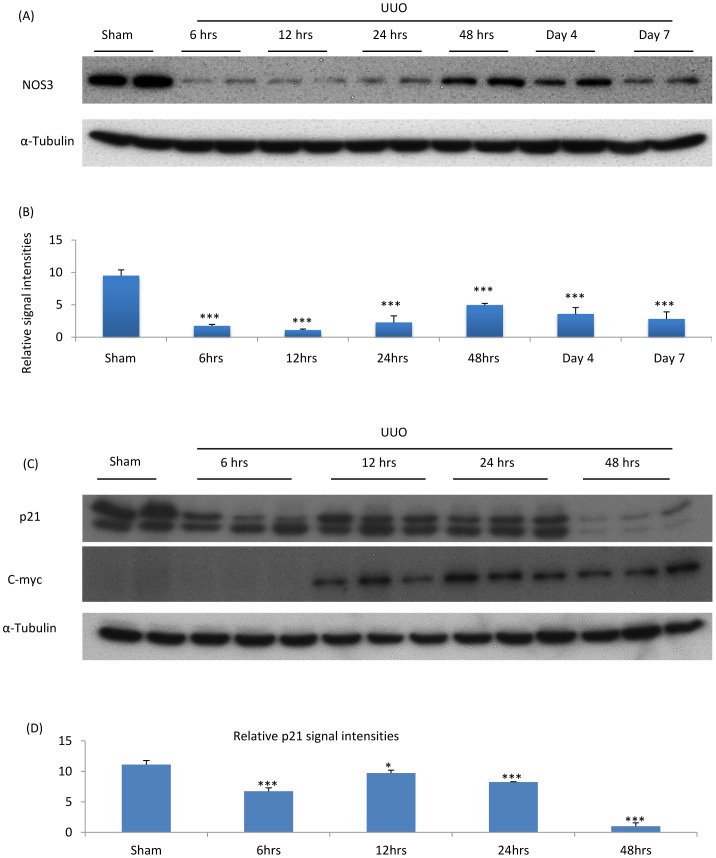
Expression of NOS3, c-myc and p21 Cip1 following unilateral ureteral obstruction (UUO). (A) Western blotting of NOS3 in sham operated and UUO kidneys; (B) Quantification of NOS3 expression relative to α-Tubulin; (C) Western blotting of C-myc and p21Cip1; and (D) Quantification of p21Cip1 expression relative to α-Tubulin. Data are mean ±SD from groups of 5 mice and analysis by one-way ANOVA with post hoc analysis with Tukey's multiple comparison test. ***p<0.001 versus sham operated group.

Endothelial dysfunction was evident as early as 6 hr after unilateral ureteral obstruction (UUO) on the basis of an 85% reduction of NOS3 protein levels compared to sham operated controls, followed by a partial recovery of NOS3 expression, although NOS3 levels remained below normal for the entire 7 days following UUO (Fig 1A&B). Expression of c-myc became evident at 12 hr after UUO while there was a modest reduction in p21 levels at 6 hr and a substantial reduction in p21 levels at 48 hr after UUO (Fig 1C&D), consistent with previous studies showing that proliferation of interstitial myofibroblasts and tubular cells becomes evident at 48 hrs after UUO [34]. Thus, down-regulation of NOS3 expression is an early event following UUO and precedes fibroblast proliferation and the development of interstitial fibrosis.

### Phosphorylation of the Smad3 Linker Region Precedes that of the C-Terminal Domain in the Obstructed Kidney

**Figure 2:**
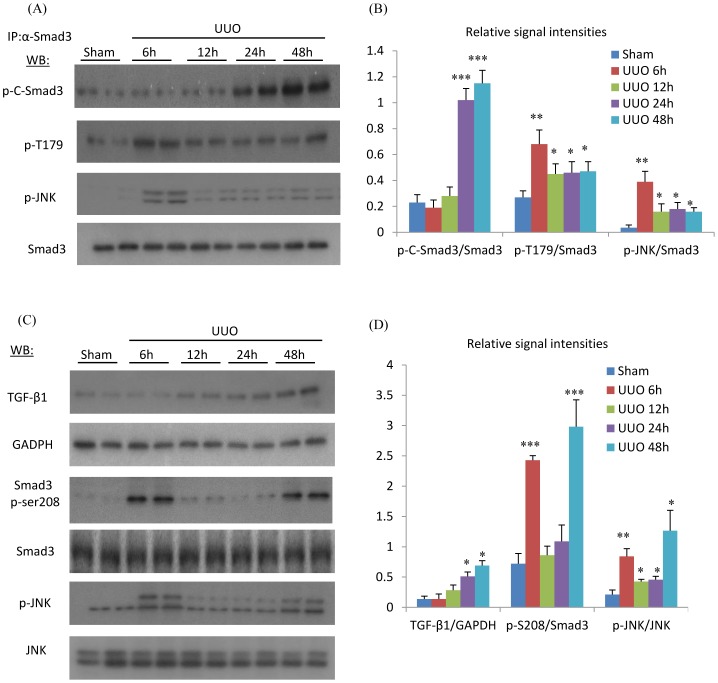
Phosphorylation of JNK, the Smad3 linker region (T179 and S208) and the Smad3 C-terminal domain following unilateral ureteral obstruction (UUO) and in sham operated controls. (A) Immunoprecipitation (IP) of Smad3 followed by Western Blotting (WB) identified phosphorylation of the Smad3 C-terminal domain (p-C-Smad3), phosphorylation of the Smad3 linker region (p-T179) in the UUO kidney. IP of Smad3 also pulled down phosphorylated JNK (p-JNK). Detection of total Smad3 confirms equal efficiency of Smad3 precipitation. The results are quantified in (B). (C) Direct WB of kidney lysates was used to analyse protein levels of TGF-β1, phosphorylation of Smad3 linker region at S208 (p-S208) and total p-JNK. The results are quantified in (D). Data are mean ±SD from groups of 5 mice and analysis by one-way ANOVA with post hoc analysis with Tukey's multiple comparison test. *p<0.05, **p<0.01, ***p<0.001 versus sham operated group.

Analysis of kidney tissue by immunoprecipitation and Western blotting showed that an increase in Smad3 C-terminal phosphorylation was first evident 24 hr after UUO (Fig 2A&B), together with an increase in kidney TGF-β1 mRNA levels (Fig 2C&D). In contrast, increased phosphorylation of the Smad3 linker region at T179 and S208 was seen at 6 hr after UUO (Fig 2A–D), identifying a different kinetics in the phosphorylation of the linker and C-terminal regions of Smad3. Activation (phosphorylation of the activation loop) of the c-Jun amino terminal kinase (JNK) was also increased at 6 hr after UUO (Fig 2C&D). Furthermore, immunoprecipitation identified a direct interaction between active JNK and Smad3 at 6 hr after UUO (Fig 2A&B), suggesting a role for JNK in phosphorylating the Smad3 linker region in the development of renal fibrosis.

### NOS3 Deficiency Augments Peritubular Capillary (PTC) Loss, Renal Fibrosis and Phosphorylation of the Smad3 Linker Region in the Obstructed Kidney

**Figure 3:**
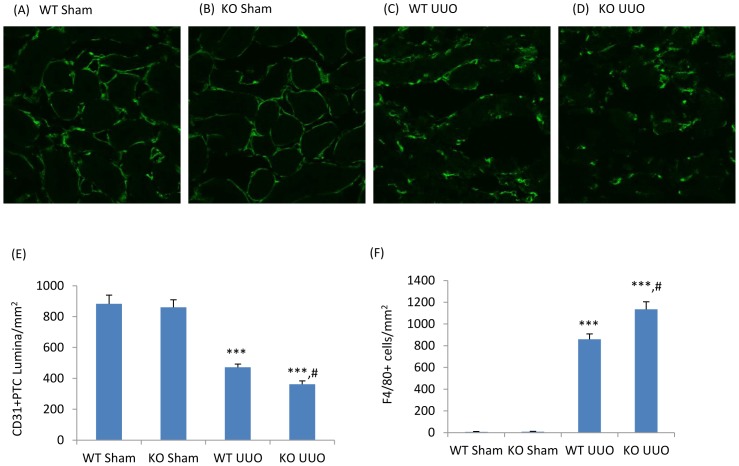
Peritubular capillaries (PTC) and macrophage infiltration in unilateral ureteral obstruction (UUO). Confocal microscopy identifies CD31^+^ (green) PTC endothelial cells in wild type (WT, A&C)) and NOS3 knockout (KO, B&D) mouse kidneys 7 days after sham (A&B) or UUO surgery (C&D). In UUO kidney, PTCs appear compressed and misshapen (C&D). Quantification of CD31+ PTC lumina/mm^2^ (E) and F4/80+ macrophages infiltration (F). Data are mean ±SD from groups of 5 mice and analysis by one-way ANOVA with post hoc analysis with Tukey's multiple comparison test. ***p<0.001 versus sham operated group. #*p*<0.05 versus WT operated group. Magnification, ×600 in A to D.

**Figure 4:**
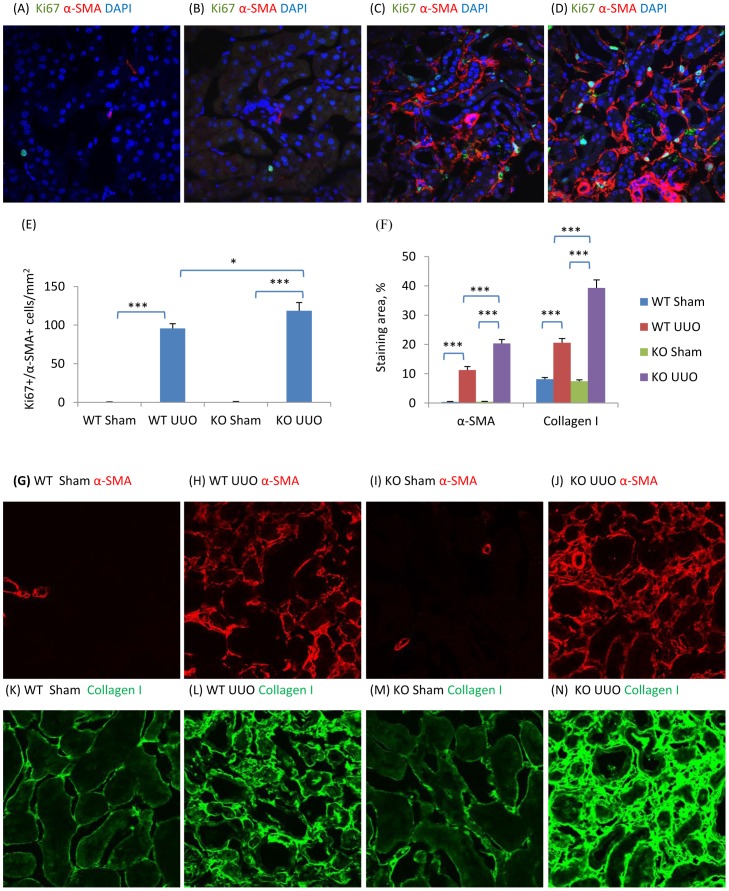
NOS3 deficiency promotes renal interstitial fibrosis on day 7 following unilateral ureteral obstruction (UUO). (A–D) Confocal microscopy identification of Ki67 (green), α-smooth muscle actin (α-SMA, red) and nuclear staining DAPI (blue) in wild type (WT) mouse kidney (A&C) or NOS3-/- (KO) kidney (B&D) following a sham operation (A&B) or UUO surgery (C&D). Quantification of: (E) Ki67+α-SMA+DAPI+ cells/mm^2^, and; (F) the area of α-SMA and collagen I staining in WT and KO mouse kidney in sham operated and UUO kidneys. (G–N) Confocal microscopy identifying α-SMA (G–J, red) and collagen I (K–N, green) staining in WT (G, H, K and L) or KO (I, J, M and N) mouse kidneys with sham (G, I, K and M) or UUO (H, J, L and N) surgery. Data are mean ±SD from groups of 5 mice and analysis by one-way ANOVA with post hoc analysis with Tukey's multiple comparison test. *p<0.05, ***p<0.001 versus sham operated control.

**Figure 5:**
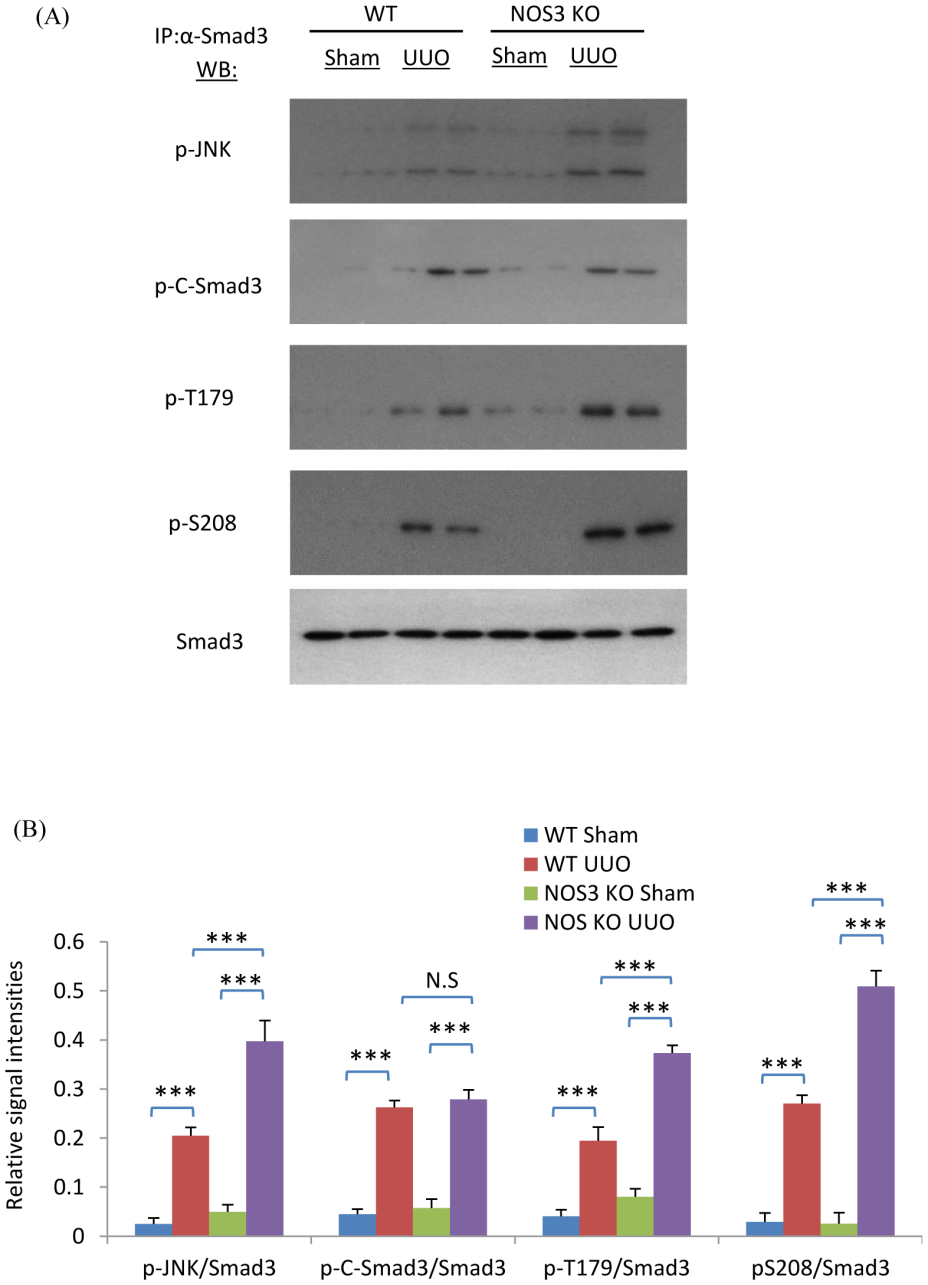
NOS3 deficiency enhanced phosphorylation of JNK and the Smad3 linker region on day 7 following unilateral ureteric obstruction (UUO), but not the Smad3 C-terminal domain. (A) Immunoprecipitation (IP) of Smad3 followed by Western Blotting (WB) identified phosphorylation of the Smad3 C-terminal domain (p-C-Smad3) and phosphorylation of the Smad3 linker region (p-T179 and p-S208) in the UUO kidney. In addition, Smad3 IP pulled down phosphorylated JNK (p-JNK). Detection of total Smad3 confirms equal efficiency of Smad3 precipitation. (B) Quantification of blotting results. Data are mean ±SD from groups of 5 mice and analysis by one-way ANOVA with post hoc analysis with Tukey's multiple comparison test. *p<0.05, ***p<0.001 versus sham operated control.

To investigate the role of decreased NOS3 levels in the development of renal interstitial fibrosis, NOS3-/- mice were examined in the UUO model. Confocal microscopy demonstrated that UUO induced a significant loss of CD31+ PTC compared to the sham operation kidney while NOS3 deficiency further exacerbated the loss of PTC (Fig 3A–E). In addition, NOS3 deficiency significantly augmented the F4/80+ macrophage infiltrate (Fig 3F). Myofibroblast proliferation (α-SMA+Ki67+ cells) was augmented in NOS3-/- compared to wild type (WT) mice (Fig 4A–E). This was associated with enhanced α-SMA+ myofibroblast accumulation and collagen I deposition on day 7 UUO (Fig 4F–N). Further analysis identified enhanced Smad3 phosphorylation in the linker region (T179 and S208) and augmented binding of active JNK to Smad3 in the NOS3-/- UUO kidney, whereas Smad3 C-terminal phosphorylation was not different between NOS3-/- and WT UUO (Fig 5A&B).

### Resolvin D1 Prevents Down-Regulation of NOS3 and Reduces Smad3 Linker Phosphorylation and Renal Fibrosis in the Obstructed Kidney

**Figure 6:**
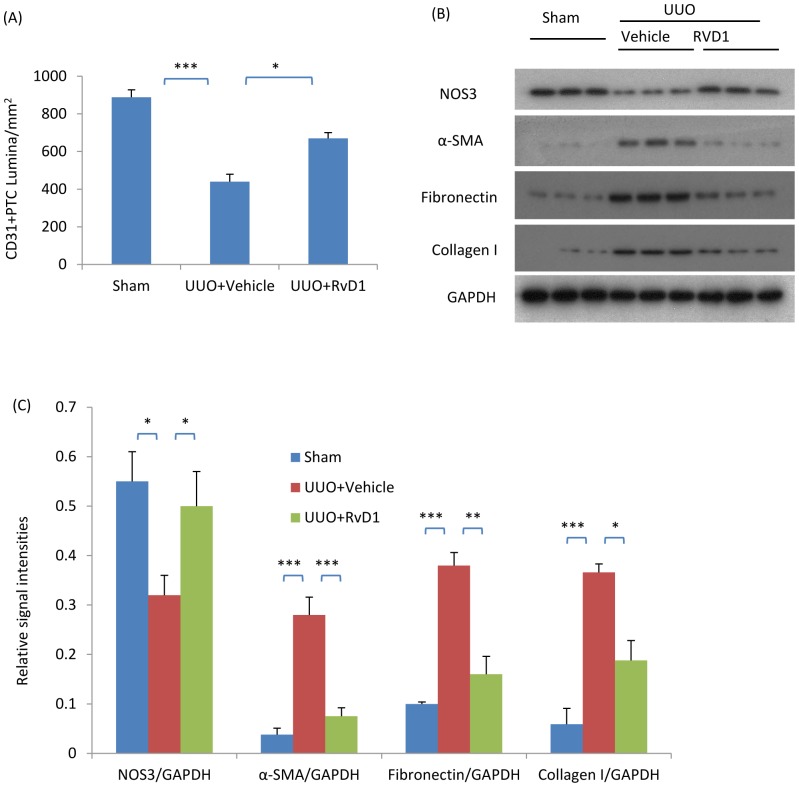
RvD1 reduced renal interstitial fibrosis in the obstructed kidneys of wild type mice. Mice underwent sham or unilateral ureteral obstruction (UUO) surgery. Two days after UUO surgery, mice received intraperitoneal injections of RvD1 for another two days and were killed on day 4. (A) Quantification of CD31+ peritubular capillary (PTC) lumina; (B) Western blotting analysis of kidney protein levels of NOS3, α-smooth muscle actin (α-SMA), fibronectin, collagen I and GAPDH. (C) Quantification of the relative abundance of NOS3, α-SMA, collagen I and GAPDH. Data are mean ±SD from groups of 6 mice and analysis by one-way ANOVA with post hoc analysis with Tukey's multiple comparison test. *p<0.05, **p<0.01, ***p<0.001.

**Figure 7:**
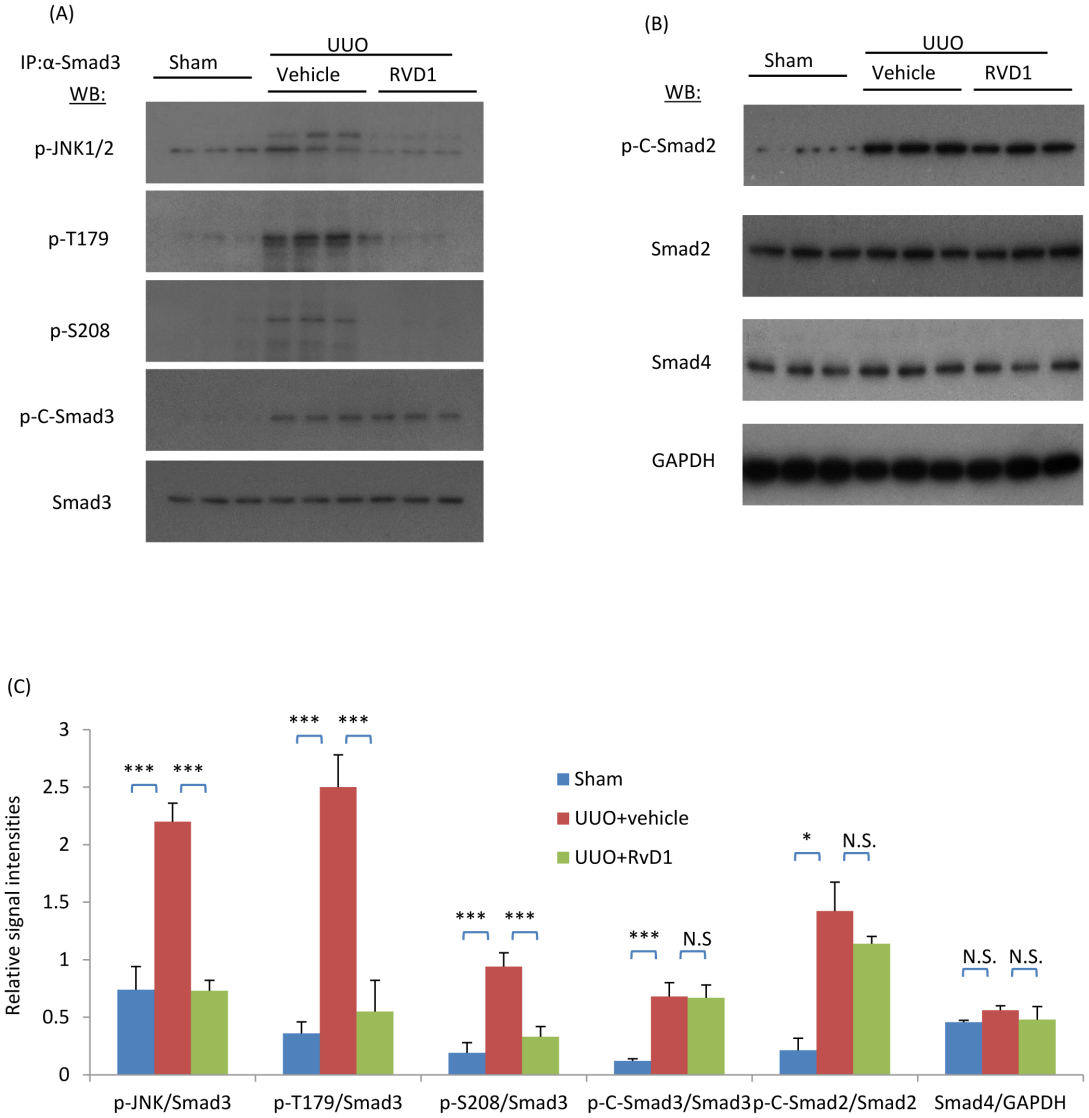
RvD1 inhibited phosphorylation of JNK and the Smad3 linker region but not Smad3 c-terminal phosphorylation in the obstructed kidney. Mice underwent sham or unilateral ureteral obstruction (UUO) surgery. Two days after UUO surgery, mice received intraperitoneal injections of RvD1 for another two days and were killed on day 4. (A) Immunoprecipitation (IP) of Smad3 followed by Western Blotting (WB) identified phosphorylation of the Smad3 C-terminal domain (p-C-Smad3) and linker region (p-T179 and p-S208) following unilateral ureteric obstruction (UUO) compared to the sham operated control. In addition, Smad3 IP pulled down phosphorylated JNK (p-JNK). (B) Western blot identifying phosphorylation of the Smad2 C-terminal domain (p-C-Smad2), total Smad2, total Smad4 and GAPDH. (C) Quantification of the relative levels of abundance of p-JNK, p-T179, p-S208, p-C-Smad3 compared to total Smad3, p-C-Smad2 compared to total Smad2 and total Smad4 compared to GAPDH. Data are mean ±SD from groups of 6 mice and analysis by one-way ANOVA with post hoc analysis with Tukey's multiple comparison test. *** *p*<0.001; N.S., not significant.

We have previously shown that Resolvin D1 (RvD1) treatment can suppress fibroblast proliferation and renal fibrosis in the UUO model [34]. In studies of these archival tissues, confocal microscopy demonstrated that RvD1 treatment reduced the loss of CD31+ PTC lumina (Fig 6A) while Western blotting confirmed the reduction in α-SMA, fibronectin and collagen I proteins following RvD1 treatment over days 2 to 4 UUO in WT mice (Fig 6B&C). In addition, we identified that RvD1 treatment prevented the reduction in NOS3 protein levels in the UUO kidney (Fig 6B&C). Further analysis of these tissues showed that RvD1 substantially inhibited phosphorylation of the Smad3 linker region and the formation of complexes of active JNK with Smad3, although phosphorylation of the C-terminal domain of Smad2 and 3 and total Smad4 levels were not affected (Fig 7).

### Conditioned Media from L-NAME Treated Endothelial Cells Enhances Fibroblast Proliferation, Collagen Production and Smad3 Linker Phosphorylation

**Figure 8:**
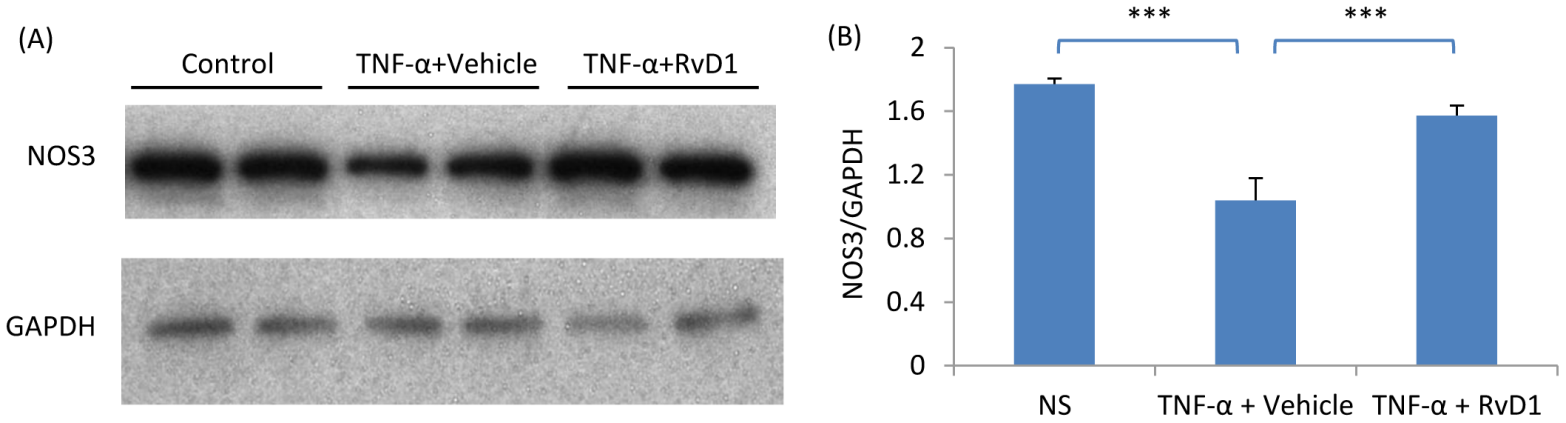
RvD1 prevents TNF-α induced down-regulation of NOS3 expression in mouse endothelial cells. (A) Western blotting of NOS3 levels in mouse microvascular endothelial cells (MMEC) cultured under control conditions, with 10 ng/ml TNF-α or with TNF-α plus 4 ng/ml RvD1 for 12 hr. (B) Quantification of NOS3 expression relative to GAPDH. Data are mean ±SD and analysis by one-way ANOVA with post hoc analysis with Tukey's multiple comparison test. *** *p*<0.001.

**Figure 9:**
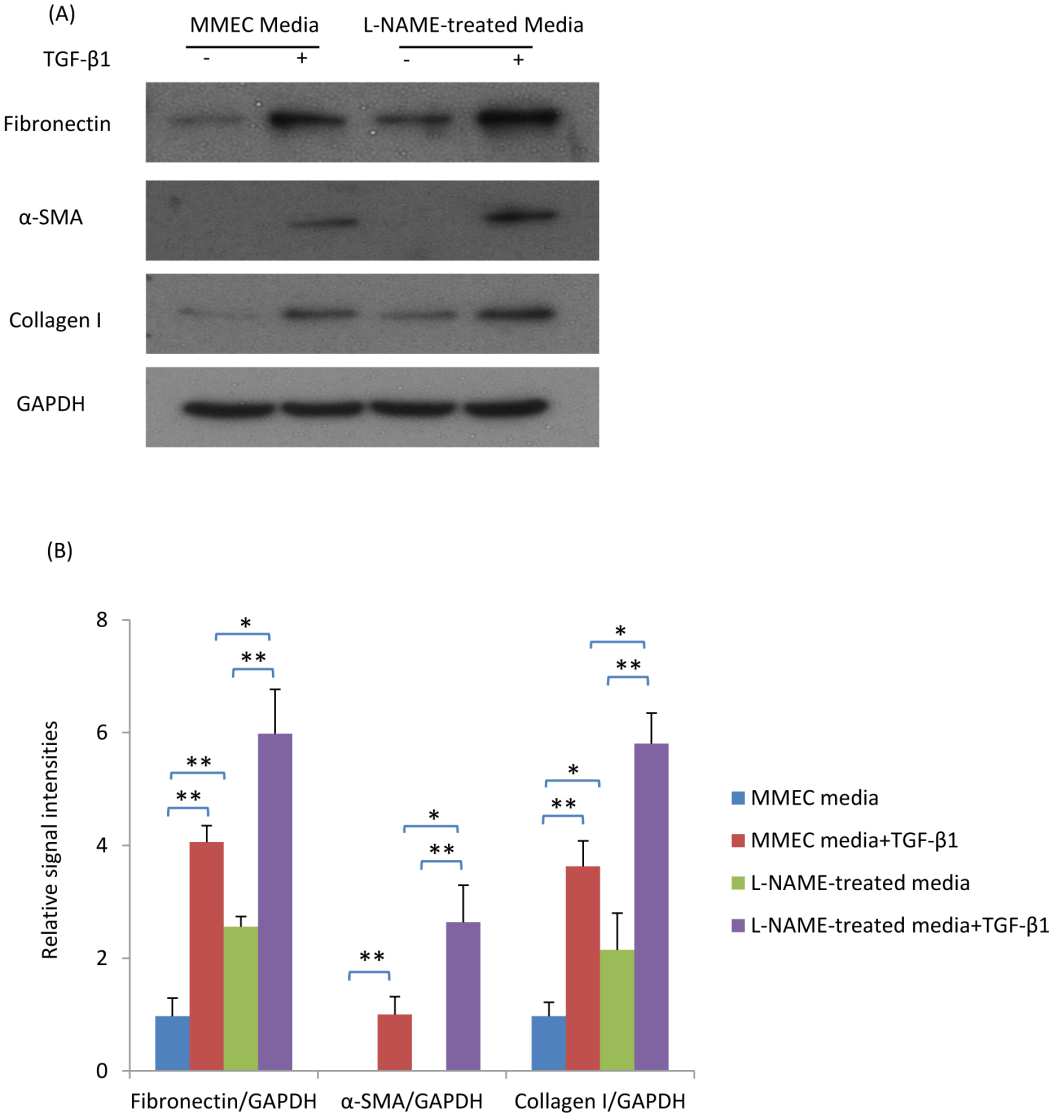
L-NAME-treated media induces a pro-fibrotic response in renal fibroblasts (NRK49F cells). (A) Western blot showing expression of fibronectin, α-smooth muscle actin (α-SMA), collagen I and GAPDH in NRK49F cells cultured with L-NAME-treated or control MMEC media in the presence or absence of TGF-β1 stimulation for 24 hrs. (B) Quantification of the relative expression of fibronectin, α-SMA and collagen I compared to GAPDH. Data are mean ±SD and analysis by one-way ANOVA with post hoc analysis with Tukey's multiple comparison test. *p<0.05, **p<0.01. Experiments were repeated at least three times.

**Figure 10:**
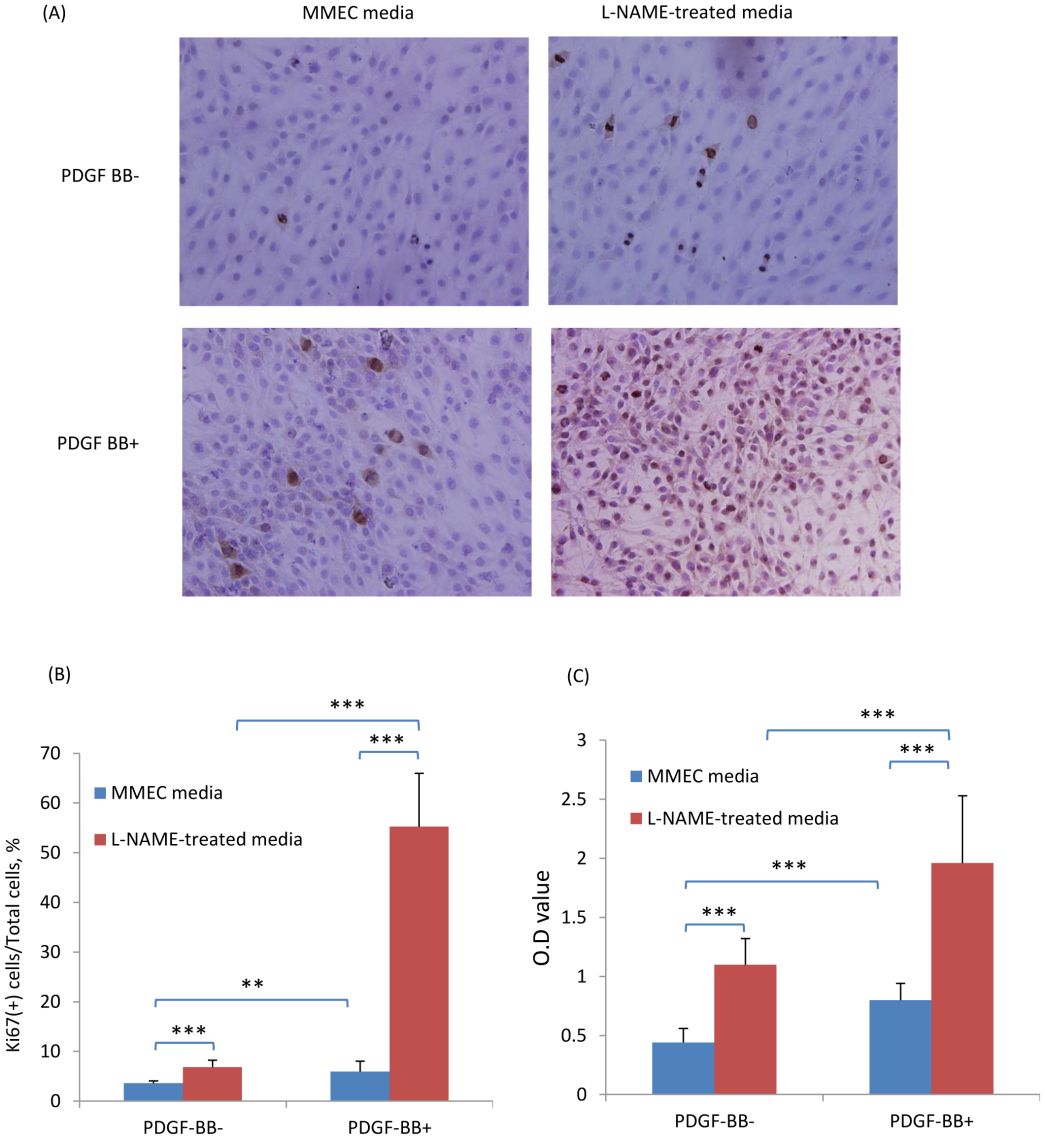
L-NAME-treated media enhances PDGF-BB-induced proliferation of renal fibroblasts (NRK49F cells). Sub-confluent NRK49F cells were incubated in L-NAME-treated or control MMEC media in the presence or absence of PDGF-BB stimulation for 24 hr. (A) Immunostaining of Ki67 (brown) with nuclear staining (blue) in NRK49F cells. (B) Quantification of the percentage of NRK49F cells positive for Ki67 immunostaining. (C) Quantification of cell proliferation using incorporation of bromodeoxyuridine (Brdu). Data are mean ±SD and analysis by one-way ANOVA with post hoc analysis with Tukey's multiple comparison test. **p<0.01, ***p<0.001. Experiments were repeated at least three times.

**Figure 11:**
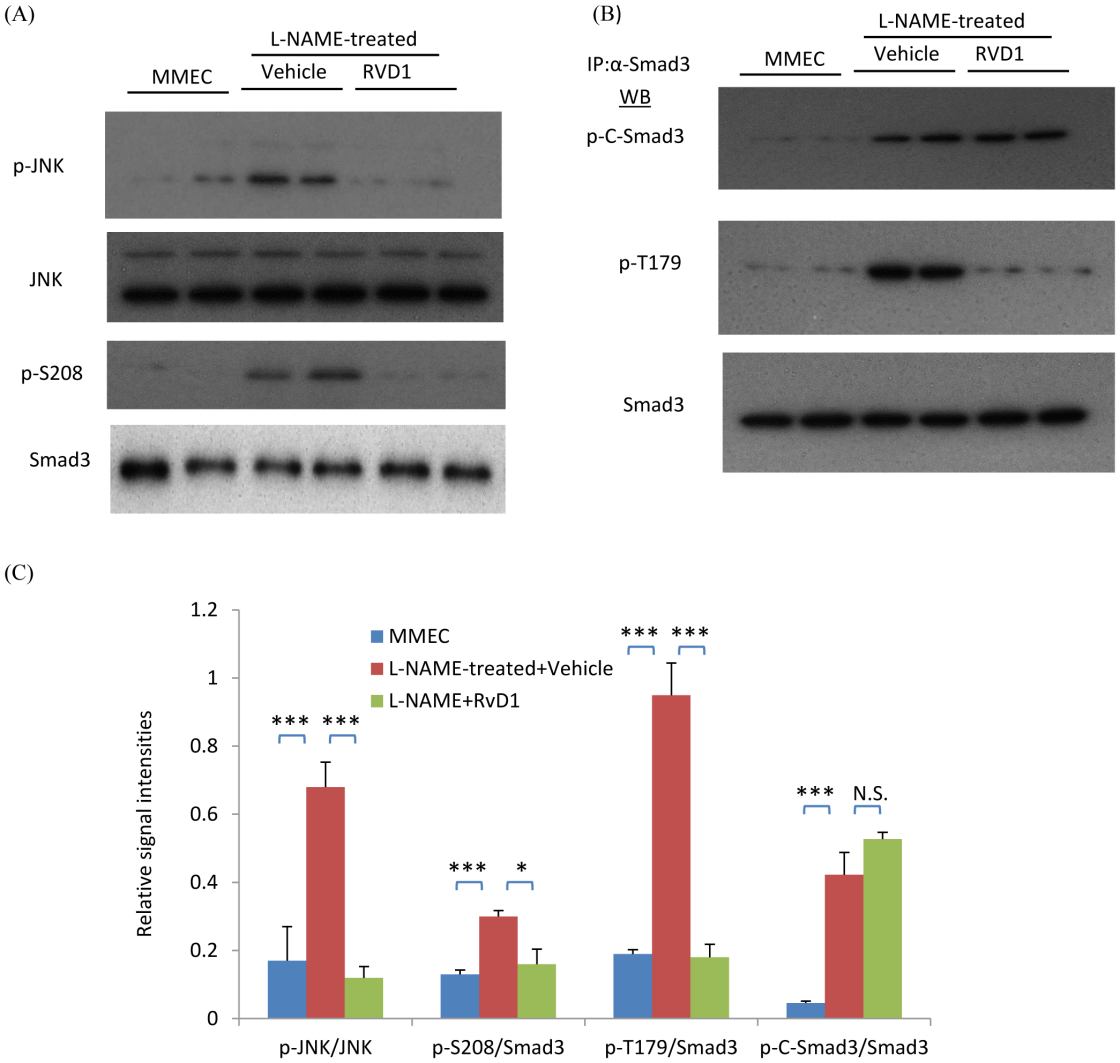
L-NAME-treated media induces phosphorylation of JNK and the Smad3 linker region in renal fibroblasts (NRK49F cells) which is inhibited by RvD1. (A) Western blot (WB) showing phosphorylation of JNK and p-S208 in the Smad3 linker region in NRK49F cells incubated with L-NAME conditioned MMEC media which is inhibited by RvD1. (B) Immunoprecipitation (IP) of Smad3 followed by WB identified phosphorylation of the Smad3 C-terminal domain (p-C-Smad3) and linker region (p-T179) following culture of NRK49F cells in L-NAME conditioned MMEC media which is inhibited by RvD1. (C) Quantification of phosphorylation of JNK and the different sites on Smad3. Data are mean ±SD and analysis by one-way ANOVA with post hoc analysis with Tukey's multiple comparison test. *p<0.05, ***p<0.001, N.S., not significant. Experiments were repeated at least three times.

To investigate how a lack of nitric oxide production by endothelial cells can affect fibroblast responses, mouse microvascular endothelial cells (MMECs) were treated with the nitric oxide synthase inhibitor, L-NAME, and the effects of MMEC conditioned media assessed upon renal fibroblasts (NRK49F cells). First, we confirmed that MMECs express high levels of NOS3 (Fig 8). Compared with control MMEC media, L-NAME-treated media (filtered to remove residual L-NAME) induced higher levels of fibronectin and collagen I in renal fibroblasts (Fig 9A&B). Furthermore, the up-regulation of fibronectin, α-SMA and collagen I expression in response to TGF-β1 stimulation of NRK49F fibroblasts was significantly enhanced by L-NAME-treated MMEC media (Fig 9A&B). In addition, L-NAME-treated conditioned MMEC media increased the proliferation of NRK49F fibroblasts and substantially augmented their proliferative response to PDGF-BB (Fig 10). Further analysis demonstrated that incubation of renal fibroblasts with L-NAME-treated but not control MMEC media induced phosphorylation of both JNK and the Smad3 linker region T179 and S208 within 30 min (Fig 11A–C). In contrast, both L-NAME-treated and control MMEC media induced equivalent phosphorylation of the Smad3 C-terminal domain (Fig 11B&C).

### Resolvin D1 Prevents the Pro-Fibrotic and Pro-Proliferative Effects of Conditioned Media from L-NAME Treated Endothelial Cells

**Figure 12:**
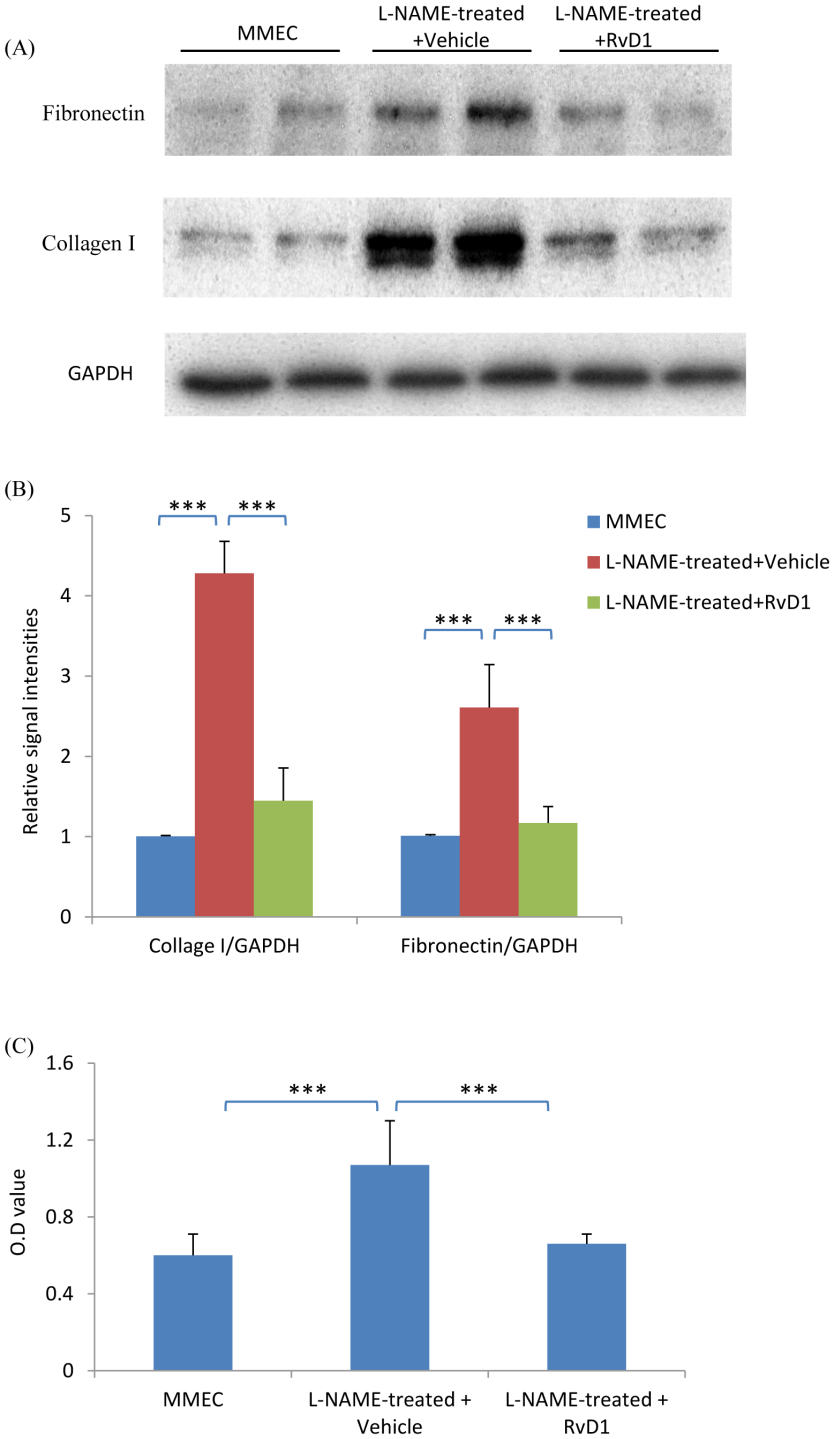
RvD1 inhibits the pro-fibrotic response induced by L-NAME-treated media in renal fibroblasts (NRK49F cells). (A) Western blot showing that incubation of NRK49F cells in L-NAME treated MMEC media for 24 hr up-regulates expression of fibronectin and collagen I which is prevented by RvD1. (B) Quantification of fibronecting and collagen I expression relative to GAPDH. ***p<0.001. (C) Quantification of bromodeoxyuridine (Brdu) uptake shows that L-NAME treated MMEC media can increase proliferation of NRK49F cells which is prevented by the addition of RvD1. Data are mean ±SD and analysis by one-way ANOVA with post hoc analysis with Tukey's multiple comparison test. ***p<0.001. Experiments were repeated at least three times.

Consistent with the *in vivo* studies, RvD1 treatment largely abolished the increased production of fibronectin and collagen I in fibroblasts incubated with L-NAME-treated MMEC media (Fig 12A&B). RvD1 also suppressed the increase in fibroblast proliferation seen with L-NAME-treated MMEC media (Fig 12C). These effects were associated with inhibition of phosphorylation of JNK and the Smad3 linker region, although phosphorylation of the Smad3 C-terminal region was not affected (Fig 11A–C).

### TGF-β1 Induced Collagen I Gene Promoter Activity is Dependent upon Smad3 Linker Phosphorylation

**Figure 13:**
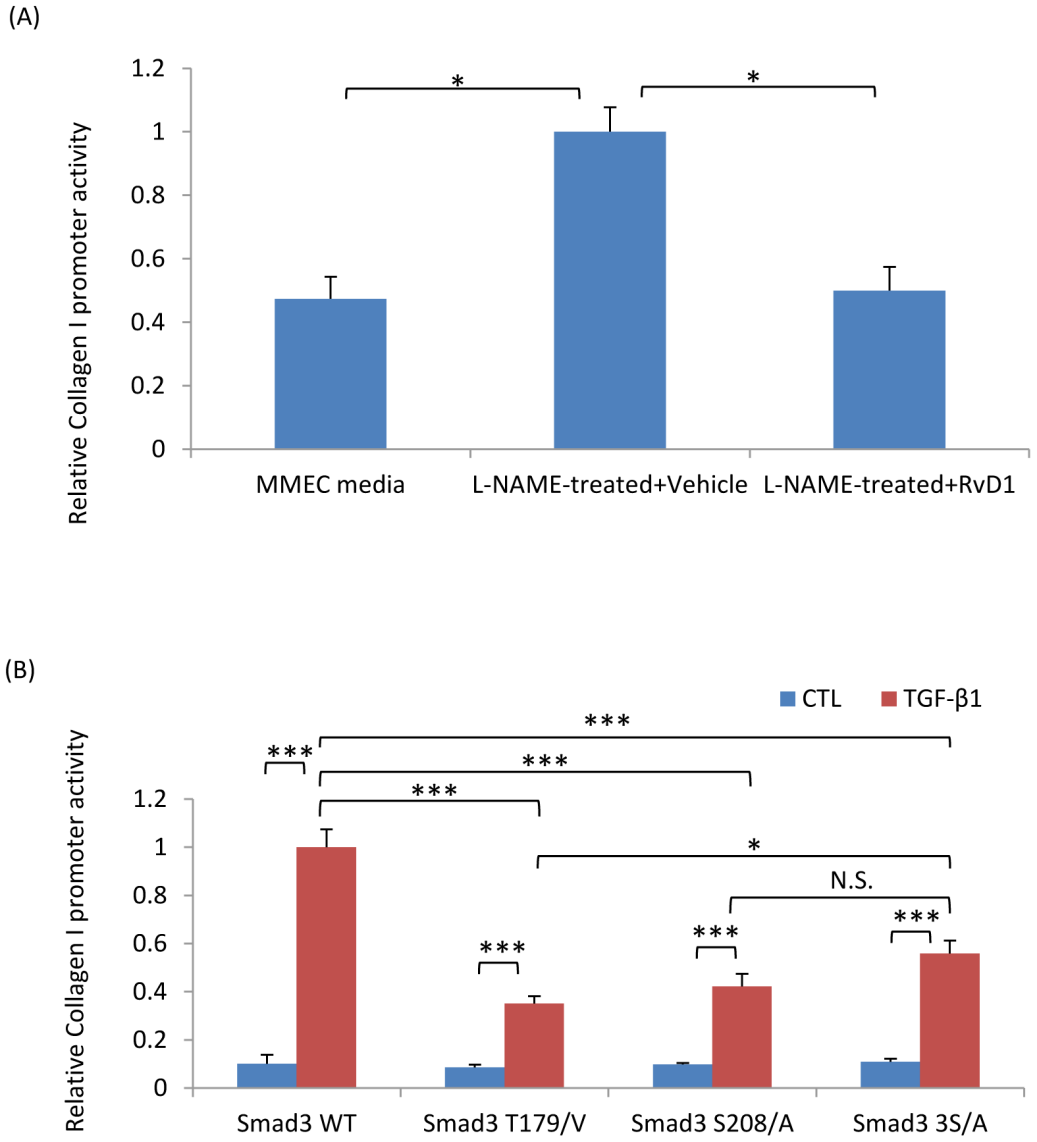
Regulation of collagen I promoter activity. (A) NRK49F cells were transfected with a reporter plasmid in which luciferase production is driven by the collagen I promoter. Incubation of these cells with L-NAME treated MMEC media for 24 hr up-regulated collagen I promoter activity which was prevented by the addition of RvD1. (B) 293T cells were transfected with Smad3 plasmids containing point mutations of various phosphorylation sites plus a collagen I promoter plasmid. Twenty-four hours later, cells were cultured with or without TGF-β1 and luciferase activity measured 24 hr later. Data are mean ±SD and analysis by one-way ANOVA with post hoc analysis with Tukey's multiple comparison test. * *p*<0.05, ***p<0.001, N.S., not significant. Experiments were repeated at least three times.

Consistent with studies of renal fibroblasts, 293T cells transfected with a collagen I promoter-driven luciferase plasmid showed a significant increase in collagen I promoter activity when incubated with L-NAME-treated compared to control MMEC media (Fig 13A). This increase in collagen I promoter activity was prevented by RvD1 (Fig 13A). To establish the functional link between Smad3 linker region phosphorylation and the Smad3-dependent pro-fibrotic response, a series of Smad3 constructs with point mutations were transfected into 293T cells. Mutation of T179/V or S208/A in the Smad3 linker region substantially reduced TGF-β1 induced collagen I promoter activity (Fig 13B). A significant reduction in collagen promoter activity was also evident with three S/A point mutations in the Smad3 terminal region (Fig 13B).

## Discussion

This study has identified that down-regulation of eNOS expression is an early event following unilateral ureteric obstruction and that a lack of eNOS gene expression enhances fibroblast proliferation and increases collagen production in the obstructed kidney. This is postulated to operate via an increase in Smad3 linker region phosphorylation through enhanced JNK activity.

A dramatic reduction in eNOS expression was an early response to kidney injury induced by unilateral ureteric obstruction. This preceded the onset of fibroblast proliferation and collagen production which is first evident on day 2 in this model [34]. Direct evidence that a lack of eNOS expression promotes fibrosis came from the finding of exacerbated renal fibrosis in the obstructed kidney of eNOS-/- mice. This is consistent with, and extends, previous studies in which administration of the non-selective nitric oxide synthase inhibitor L-NAME augments fibrosis in the UUO model, whereas administration of the nitric oxide synthase substrate L-arginine suppressed interstitial fibrosis in this model [38]–[40]. Our findings are also consistent with studies demonstrating that eNOS-/- mice exhibit exacerbated glomerular fibrosis in models of diabetic nephropathy and adriamycin-induced nephropathy [6], [41]–[43]. Conversely, NOS3 over expression by adenoviral gene delivery reduces renal dysfunction, proteinuria and fibrosis in the 5/6^th^ kidney nephrectomy model [44], and corrects endothelial dysfunction in angiotensin II-induced hypertensive rats [45].

A number of mechanisms have been proposed for the exacerbation of kidney injury in NOS3-/- mice, including higher systemic and glomerular blood pressure, and peritubular capillary endothelial cell loss which impairs oxygen delivery to the tubulointerstitial compartment [46]–[48]. In particular, the vasoconstrictor angiotensin II is known to promote renal fibrosis in the UUO model [49]. In addition, eNOS-derived NO counter-regulates angiotensin II induced contraction of microperfused renal afferent arterioles [50], while angiotensin II blockade can significantly increased renal blood flow during the initial stages of acute ureter obstruction [51]. Furthermore, the exacerbation of atherosclerotic lesions in NOS3-/-ApoE-/- mice could be attenuated by angiotensin II blockade [46]. Other factors, such as an increase in endothelin-1[52] or von Willebrand Factor [53] may also be involved in this augmented kidney injury. Nitric oxide inhibits the proliferation of vascular smooth muscle cells [54] while its precursor, L-arginine, reduces endothelin-1-induced proliferation in mesangial cells [55]. However, the mechanism linking a loss of NOS3 expression to increased renal fibrosis is most likely to involve the central TGF-β1/Smad3 pro-fibrotic signalling pathway [56], [57]. We examined whether this pathway is altered in the setting of reduced or deleted eNOS expression. A clear increase in phosphorylation of the SSXS motif in the Smad3 C-terminal domain was evident in the obstructed kidney and presumably plays an important role in the fibrotic response to unilateral ureteric obstruction. However, the mechanism most closely associated with down-regulation of eNOS expression was phosphorylation of the Smad3 linker region. Within 6 hr of ureter obstruction there was increased Smad3 linker region phosphorylation in association with a marked reduction of eNOS expression and this preceded Smad3 C-terminal phosphorylation. Similarly, eNOS-/- mice showed enhanced Smad3 linker region phosphorylation without a change in C-terminal phosphorylation. Evidence of a direct link between these two events came from studies in which conditioned media from endothelial cells cultured in the presence of a nitric oxide synthase inhibitor caused a selective increase in Smad3 linker region phosphorylation in association with increased fibroblast proliferation and collagen production. This is likely to be an indirect action of secreted proteins by the treated endothelial cells since the media was extensively dialysed against a 10 kDa membrane to remove L-NAME. The exact components in the L-NAME conditioned media that promoted fibroblast proliferation and collagen production are the focus of ongoing studies. Furthermore, our in vitro studies examining Smad3 driven collagen promoter activity verified that mutation of the phosphorylation sites in the linker region strongly inhibits TGF-β1 induced collagen production, while previous studies have shown that mutation of phosphorylation sites in the linker region to glutamic acid (thereby mimicking linker region phosphorylation) augments Smad3 driven collagen production *in vitro* [19]. Indeed, while increased Smad3 linker phosphorylation has been described in cancer, this study is the first report of increased Smad3 linker region phosphorylation in kidney fibrosis. It also provides *in vivo* and *in vitro* evidence for a pro-proliferative and pro-fibrotic action of Smad3 linker region phosphorylation, which may help to clarify the contradictory findings described in different cancer cell lines [25], [26]. These findings also substantially extend a previous study showing that aortas from eNOS-/- mice exhibit enhanced basal and TGF-β1 induced collagen type I expression and that endothelial cells from these animals showed increased Smad2/3 phosphorylation [58].

The function of Smad3 depends on extensive interactions (cross-talk) with other signalling pathways [59]. While the TGF-β-receptor I directly phosphorylates the SSXS motif in the Smad3 C-terminal domain, a range of other kinases such as mitogen-activated protein kinases (JNK, ERK, p38), protein kinase B (Akt) and cyclin-dependent kinases are involved in the phosphorylation of sites in the Smad3 linker region. Thus, there is considerable scope for a range of signalling pathways to positively or negatively regulate Smad3 phosphorylation and thus transcription activity [19]–[26]. We identified JNK as a likely candidate for the increase Smad3 linker region phosphorylation in the obstructed kidney based on several findings. First, increased JNK activation was coincident with increased linker region phosphorylation at 6 hr following unilateral ureteric obstruction. Second, the increase in linker phosphorylation in eNOS-/- mice was also associated with increased JNK activation. Third, immunoprecipitation experiments demonstrated an increase in the physical association of activated JNK with Smad3. Fourth, the ability of conditioned media from L-NAME treated endothelial cells to induce increased fibroblast proliferation and collagen production was associated with both increased JNK activation and linker region phosphorylation. In addition, JNK has previously been shown to phosphorylate the Smad3 linker region [19], [20], and blockade of JNK signalling inhibits renal fibrosis in the obstructed kidney [60], [61].

We have shown previously that RvD1 suppresses renal fibrosis and fibroblast proliferation in the UUO model with inhibition of PDGF induced fibroblast proliferation *in vitro* and *in vivo* [34]. Thus the study examined the mechanisms underlying the protective effects of RvD1 in the UUO model. It was shown, both *in vivo* and *in vitro*, that RvD1 prevented down-regulation of eNOS expression and suppressed activation of JNK and Smad3 linker phosphorylation without affecting Smad3 C-terminal phosphorylation. This finding supports the postulate that JNK mediated Smad3 linker region phosphorylation is an important mechanism by which reduced eNOS expression promotes renal fibrosis. The demonstration that RvD1 can inhibit TNF-α-induced activation of endothelial cells is consistent with the ability of resolvins to modulate acute inflammation [31]–[33]; however, this is the first report that a resolvin can inhibit JNK activation. A second potential mechanism by which RvD1 may have suppressed Smad3 linker phosphorylation is through suppression of fibroblast proliferation, which would reduce CDK4 and CDK8 activity – two kinases capable of phosphorylating sites in the Smad3 linker region [25], [26].

In conclusion, we demonstrate that endothelial injury and Smad3 linker T179 and S208 phosphorylation precede myofibroblast proliferation and Smad3 C-terminal phosphorylation in the obstructed kidney. RvD1 administration reduced endothelial injury, Smad3 linker T179 and S208 activation and renal fibrosis without inhibition of Smad3 C-terminal phosphorylation. Thus, protection of endothelial cells or inhibition of the JNK-Smad3 interaction may be strategies for the treatment of kidney fibrosis.
